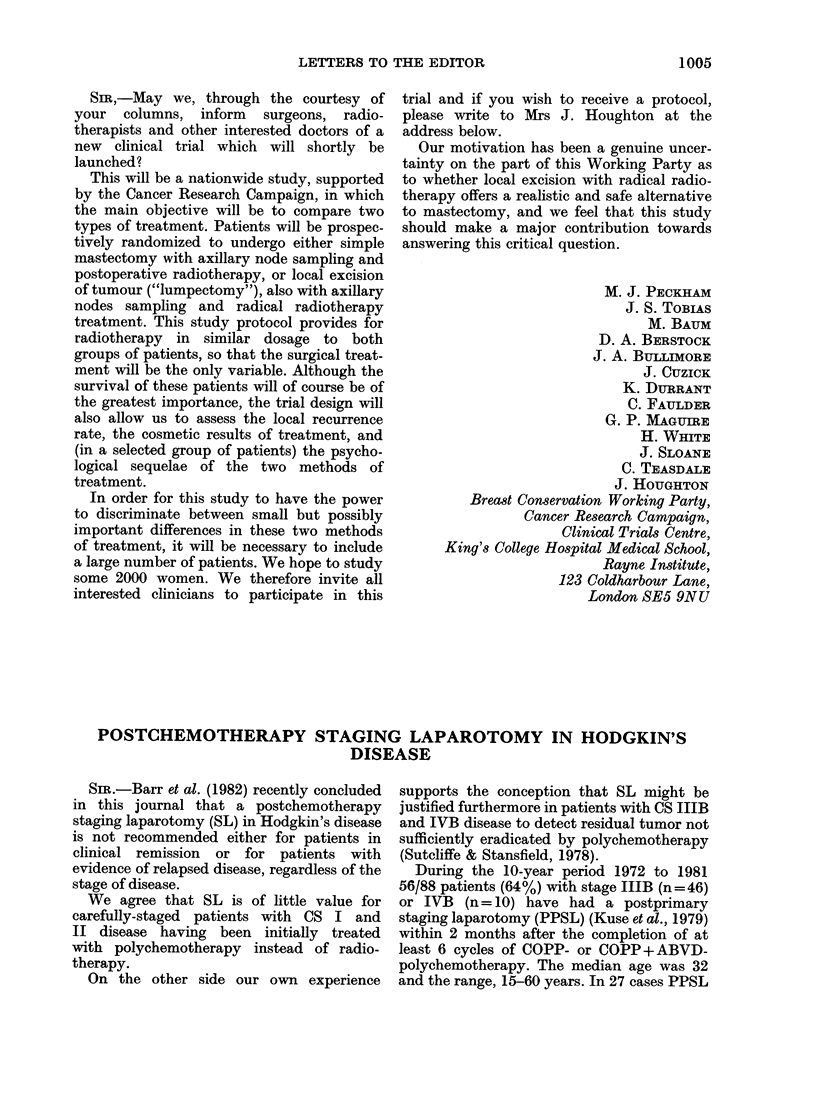# Replay to letter by Drs Gillis and Hole

**Published:** 1982-12

**Authors:** M. J. Peckham, J. S. Tobias, M. Baum, D. A. Berstock, J. A. Bullimore, J. Cuzick, K. Durrant, C. Faulder, G. P. Maguire, H. White, J. Sloane, C. Teasdale, J. Houghton


Sir,—May we, through the courtesy of your columns, inform surgeons, radiotherapists and other interested doctors of a new clinical trial which will shortly be launched?

This will be a nationwide study, supported by the Cancer Research Campaign, in which the main objective will be to compare two types of treatment. Patients will be prospectively randomized to undergo either simple mastectomy with axillary node sampling and postoperative radiotherapy, or local excision of tumour ("lumpectomy"), also with axillary nodes sampling and radical radiotherapy treatment. This study protocol provides for radiotherapy in similar dosage to both groups of patients, so that the surgical treatment will be the only variable. Although the survival of these patients will of course be of the greatest importance, the trial design will also allow us to assess the local recurrence rate, the cosmetic results of treatment, and (in a selected group of patients) the psychological sequelae of the two methods of treatment.

In order for this study to have the power to discriminate between small but possibly important differences in these two methods of treatment, it will be necessary to include a large number of patients. We hope to study some 2000 women. We therefore invite all interested clinicians to participate in this trial and if you wish to receive a protocol, please write to Mrs J. Houghton at the address below.

Our motivation has been a genuine uncertainty on the part of this Working Party as to whether local excision with radical radiotherapy offers a realistic and safe alternative to mastectomy, and we feel that this study should make a major contribution towards answering this critical question.

M. J. Peckham
J. S. Tobias
M. Baum
D. A. Berstock
J. A. Bullimore
J. Cuzick
K. Durrant
C. Faulder
G. P. Maguire
H. White
J. Sloane
C. Teasdale
J. Houghton
*Breast Conservation Working Party,*
*Cancer Research Campaign,*
*Clinical Trials Centre,*
*King's College Hospital Medical School,*
*Rayne Institute,*
*123 Coldharbour Lane,*
*London SE5 9NU*